# Maternal Enrichment during Pregnancy Accelerates Retinal Development of the Fetus

**DOI:** 10.1371/journal.pone.0001160

**Published:** 2007-11-14

**Authors:** Alessandro Sale, Maria Cristina Cenni, Francesca Ciucci, Elena Putignano, Sabrina Chierzi, Lamberto Maffei

**Affiliations:** 1 Scuola Normale Superiore, Pisa, Italy; 2 Institute of Neuroscience of Consiglio Nazionale delle Ricerche (CNR), Pisa, Italy; University of Washington, United States of America

## Abstract

The influence of maternal environment on fetal development is largely unexplored, the available evidence concerns only the deleterious effects elicited by prenatal stress. Here we investigated the influence of prenatal enrichment on the early development of the visual system in the fetus. We studied the anatomical development of the rat retina, by analyzing the migration of neural progenitors and the process of retinal ganglion cell death, which exerts a key role in sculpturing the developing retinal system at perinatal ages. The number of apoptotic cells in the retinal ganglion cell layer was analyzed using two distinct methods: the presence of pyknotic nuclei stained for cresyl violet and the appearance of DNA fragmentation (Tunel method). We report that environmental enrichment of the mother during pregnancy affects the structural maturation of the retina, accelerating the migration of neural progenitors and the dynamics of natural cell death. These effects seem to be under the control of insulin-like growth factor-I: its levels, higher in enriched pregnant rats and in their milk, are increased also in their offspring, its neutralization abolishes the action of maternal enrichment on retinal development and chronic insulin-like growth factor-I injection to standard-reared females mimics the effects of enrichment in the fetuses. Thus, the development of the visual system is sensitive to environmental stimulation during prenatal life. These findings could have a bearing in orienting clinical research in the field of prenatal therapy.

## Introduction

During development, the nervous system is highly plastic to environmental influence. Experience is essential during the first postnatal weeks of life, when sensory activity drives the refinement and maintenance of neural connections. The visual system has emerged as a paradigmatic model of development and plasticity of neuronal connections under the influence of the environment [1]. Recently, we showed that postnatal environmental enrichment, a condition of increased physical exercise, social interactions and sensory stimulation, results in a conspicuous acceleration of visual system development at behavioral, electrophysiological and molecular level [2]–[4].

Much less is known about the influence of the environment on the development of central nervous system during prenatal life. The only available evidence concerns the deleterious effects of prenatal stress on the embryonic development. Prenatal stress is tightly associated with growth retardation [5], [6], structural malformations [6], delayed motor development [7] and with behavioral anomalies and impaired cognitive functions at adult ages [8]–[14]. In humans, it is well known that the offspring of mothers experiencing stress during pregnancy have an increased risk of unexpected death due to structural malformations, increased frequency of spontaneous abortion, reduced weight at birth and display long-term behavioral abnormalities [14], [15].

Despite these data on the harmful effects of prenatal stress, the possibility that maternal exposure to conditions of increased sociality and sensory-motor activity could influence embryonic development remains unexplored. In the present study, we investigated this issue by analyzing whether maternal environmental enrichment during pregnancy affects the visual system development of the fetus. We found that maternal enrichment influences the anatomical and molecular development of the retina, accelerating the migration of neuronal progenitors and causing a marked increase in the rate of naturally occurring cell death, an essential developmental event until now considered to be programmed only by intrinsic signals, independently of experience. These changes were accompanied by a marked increase in insulin-like growth factor-I (IGF-I) expression in the retinas of enriched rats compared with standard reared animals. Furthermore, administration of anti-IGF-I antiserum provided to enriched mothers during late pregnancy totally prevented the acceleration of retinal development induced by environmental enrichment, while IGF-I infusions in standard pregnant females mimicked the EE effect of acceleration in the time course of both the migration and death of ganglion cells. These results suggest that maternal enrichment effects on retinal development are under the control of IGF-I.

## Results

### Acceleration of natural ganglion cell death dynamics by maternal enrichment during pregnancy

**Figure 1:**
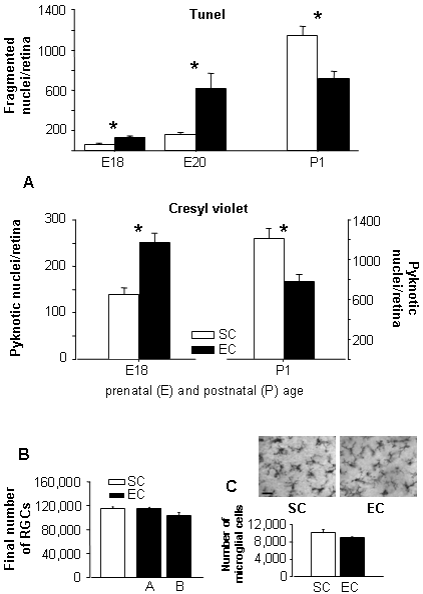
(A) Accelerated natural cell death in the RGC layer of EC rats. RGC layer apoptotic cell number in EC and SC rats, analyzed at the indicated ages with the Tunel method (top) and with cresyl violet staining of whole-mount retinas (bottom). With both methods, two-ways ANOVA showed an effect of age (*p*<0.001) and housing condition (*p*<0.05) and a significant age×housing condition interaction (*p*<0.001). Mann-Whitney rank sum test with Bonferroni correction revealed a difference between EC and SC at E18, E20 and P1 (*p*<0.001) for the tunel method, and at E18 and P1 (*p* = 0.002) for cresyl violet staining. (B) RGC number was not different between SC and EC adult rats either as estimated by calculating the 50% of total cell number in the RGC layer (A), or by subtracting the number of displaced amacrine cells remaining in the RGC layer 30 days after ipsilateral optic nerve transection from the number of cells counted in contralateral retinas (B) (*p* = 0.77 and 0.28, t-test). (C) Micrographs of RGC layer of P1 whole mount retinas labeled with B4 isolectin. No qualitative difference was detected in the shape and intensity of microglial cells between SC and EC pups. Scale bar: 20 µm. Graph: microglial cell number in the RGC layer of SC and EC rats. Mann-Whitney rank sum test showed no difference between the two groups (*p* = 0.429). Bars indicate s.e.m.

We studied the anatomical development of the retina by analyzing retinal ganglion cell (RGC) death, a process which exerts a key role in sculpturing the developing retinal system at perinatal ages in the rat. The RGC number in the mature retina is the result of a period of RGC overproduction, followed by an intense process of programmed cell death (called ‘apoptosis’). We assessed the appearance of apoptotic cells in the RGC layer using two different procedures, i.e. the counting of fragmented nuclei in coronal retinal sections reacted with the Tunel method and the counting of pyknotic cell number in cresyl violet stained whole-mount retinas. We counted apoptotic RGCs in the offspring of mothers reared in standard condition (SC) and compared the results with those obtained in the offspring of enriched (EC) mothers. We found that the temporal dynamics of RGC death were accelerated in EC animals: the number of apoptotic cells was higher in EC with respect to SC fetuses at embryonic day 18 (E18) and E20, and remarkably lower in EC compared to SC pups at postnatal day 1 (P1), when the peak of natural cell death is typically seen [16], [17] (Fig. 1*A*). We did not observe, however, any difference in RGC number between SC and EC animals, either at P1 (195240±7009 for SC, 185607±7101 for EC; *t*-test, p = 0,347) or in adulthood (Fig. 1*B*). The similar number of living RGCs in EC and SC rats could be attributable to the fact that the P1 decrement of natural cell death in EC rats could compensate for the increased number of apoptotic cells found at E18 and E20. Moreover, no differences were detected in the number and morphology of retinal microglial cells between EC and SC pups (Fig. 1*C*), indicating that the changes in the pyknotic cell number induced by maternal EE reflected a true variation in cell death rate, rather than a change in the speed of pyknotic debris' clearance.

### Maternal enrichment accelerates the migration of differentiating neural progenitors in the retina of the fetus

**Figure 2:**
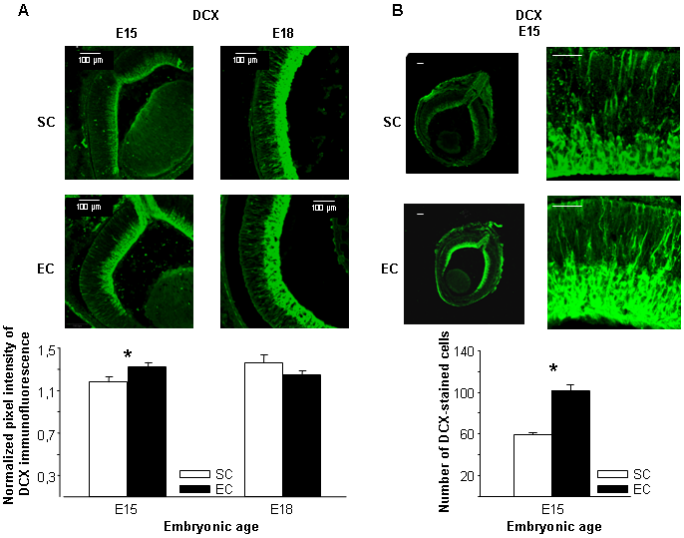
Anticipated migration of retinal neural progenitors in EC fetuses. (A, top) Micrographs of EC and SC retinal sections immunostained for double-cortin (DCX) at E15 and E18. DCX expression was increased in EC rats at E15. (A, bottom) Quantitative analysis of DCX immunofluorescence intensity in the outer retinal layers of SC and EC rats. Two-ways ANOVA showed a statistical interaction between animal age and housing condition (*p* = 0.02). A Pairwise Multiple Comparison Procedure (Holm-Sidak method) revealed that the two groups were statistically different at E15 (*p* = 0.03), but not at E18 (*p* = 0.19). (B, top) Micrographs of EC and SC retinal sections immunostained for double-cortin (DCX) at E15, acquired at 5× (left, scale bar: 100 µm) or 20× (right, scale bar: 50 µm) magnification to count the number of migrating cells. (B, bottom) Number of cells stained for DCX in the outer retinal layers of SC and EC rats. The number of DCX-labeled cells was higher in EC than in SC embryos (Mann-Whitney rank sum Test, *p*<0.05). Bars indicate s.e.m.

**Figure 3:**
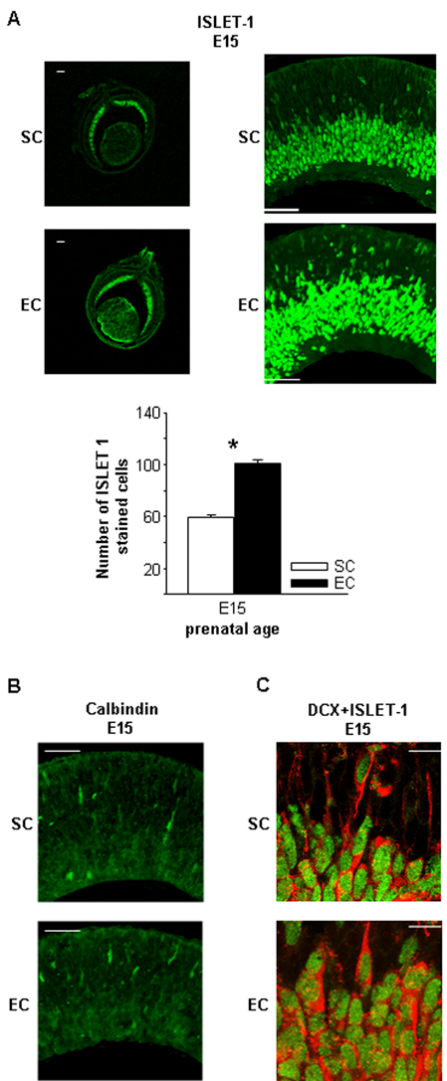
Identification of specific cell types involved in the accelerated migration of retinal cells in EC fetuses. (A, top) Micrographs of EC and SC retinal sections immunostained for ISLET-1 (a marker for ganglion and cholinergic amacrine cells) at E15, acquired at 5× (left, scale bar: 100 µm) or 20× (right, scale bar: 50 µm). (A, bottom) Number of cells stained for ISLET-1 in the outer retinal layers of SC and EC rats. The number of ISLET-1-labeled cells was higher in EC than in SC embryos (t-test, *p*<0.001). Bars indicate s.e.m. (B) Micrographs of EC and SC retinal sections immunostained for calbindin (a marker for horizontal cells) at E15, acquired at 20× (scale bar: 50 µm). The number of calbindin-labeled cells did not differ between EC and SC embryos (t-test, *p* = 0.253). (C) Micrographs of EC and SC retinal sections co-immunostained for DCX (red) and ISLET-1 (green) at E15, acquired at 60x. Scale bar: 10 µm.

We assessed whether the increased levels of cell death we found in EC pups were due to an accelerated migration of retinal neural progenitors into the RGC layer. To this purpose, E15 and E18 retinal slices from EC and SC fetuses were immunostained for double-cortin (DCX), which labels migrating cells and is a good marker of the temporal and spatial distribution of neural progenitors during the early developmental stages of the rat retina [18]. Levels of DCX immunofluorescence did not differ between EC and SC fetal retinas at E18, when a clear band of staining appeared in the RGC layer, reflecting the progressive accumulation of migrating cells during retinal maturation (Fig. 2*A*). On the contrary, we found higher levels of DCX pixel intensity immunofluorescence in the retinal outer layers of EC compared to SC animals at E15 (Fig. 2*A*). To assess whether the enhanced intensity of staining was due to an increased DCX cell number, we counted migrating cells in the region comprised from the neural progenitor layer to the RGC layer in E15 EC and SC embryos. We found that the number of migrating cells marked for DCX was significantly higher in EC with respect to SC fetuses (Fig. 2*B*). We then evaluated whether the EE effects were restricted to specific retinal cell types. Distinct populations of differentiating cell types were marked with cell-specific antibodies such as Islet-1 (a marker for ganglion and cholinergic amacrine cells), calbindin (a marker for horizontal cells) and rodopsin (a marker for photoreceptors), and the number of each differentially labeled cell type was counted in the retinal outer layers of EC and SC animals at E15. We found a higher number of cells immunoreactive for Islet-1 in EC compared to SC fetuses (Fig. 3*A*). Instead, we did not observe any difference between EC and SC embryos in the number of calbindin positive cells (Fig. 3*B*), while rodopsin was not expressed in the fetal retina of both environmental groups. It is interesting to note that the number of DCX positive cells was very similar to that of Islet-1 labeled cells, as further confirmed by a double labeling experiment in which DCX-positive cells were found to be immunoreactive also for Islet-1 (Fig. 3*C*). These results indicate that the influence of maternal enrichment on retinal structural development extends also to maturational stages prior natural cell death.

### Maternal enrichment effects on retinal development are dependent of higher levels of IGF-I

**Figure 4:**
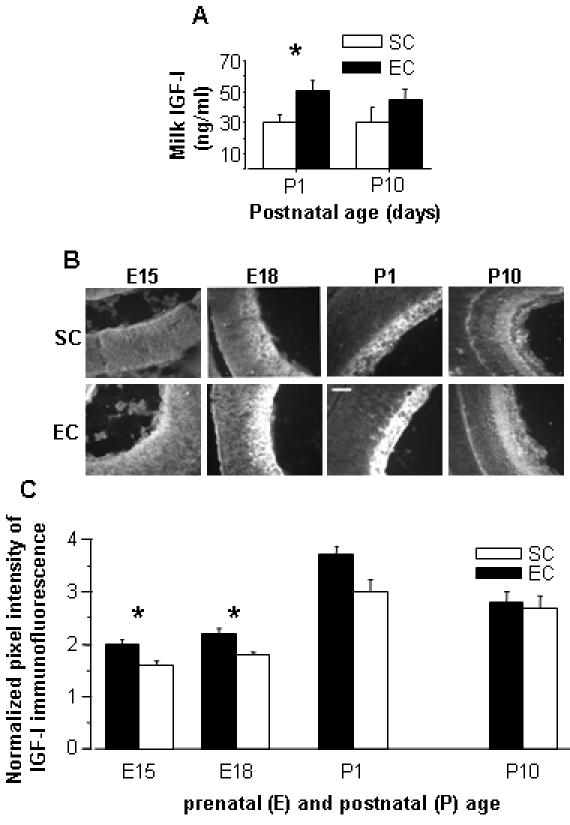
(A) Increased IGF-I concentration in the maternal milk. RIA determination of IGF-I concentration in the milk of SC and EC suckling pups: two-ways ANOVA showed a significant age×housing condition interaction (*p*<0.05). *Post-hoc* Tukey test revealed a difference at P1 (*p*<0.05), but not at P10 (*p* = 0.258) between EC and SC groups. Bars indicate s.e.m. (B–C) Enhanced IGF-I expression in the RGC layer of EC rats. (B) Micrographs of EC and SC retinal sections immunostained for IGF-I at different ages. Scale bar: 50 µm (C) Quantitative analysis of IGF-I immunofluorescence intensity in the RGC layer of SC and EC rats. Two-ways ANOVA showed an effect of age (*p*<0.001) and housing condition (*p*<0.001). t-test with Bonferroni correction revealed a statistical difference between EC and SC groups at E15 (*p* = 0.009) and E18 (*p*<0.01).

To shed light on possible molecular mechanisms mediating the influence of maternal EE on retinal development, we focused on the growth factor IGF-I, which is known to play a central role in building the cytoarchitecture of the retina [19], [20]. We first studied IGF-I in the mothers. Since it is known that differences in blood-borne IGF-I are hard to detect due to its uptake by various tissues [21], we measured IGF-I levels in the brain and in the milk of EC and SC pregnant rats. We found higher levels of IGF-I in the brain of EC compared to SC pregnant rats (data not shown) and an increase of IGF-I in the milk of EC dams in the first day postpartum (Fig. 4*A*). To investigate whether the increment of IGF-I detected in the mother was present also in the offspring, we analyzed IGF-I expression in the RGC layer from E15 to P10, when the period of RGC death is almost concluded [16]. IGF-I expression was found to be developmentally regulated in the RGC layer, progressively increasing during late embryonic life. EC animals displayed a marked increase in RGC layer levels of IGF-I at E15 and at E18, as shown in Fig. 4*B and 4C*.

**Figure 5:**
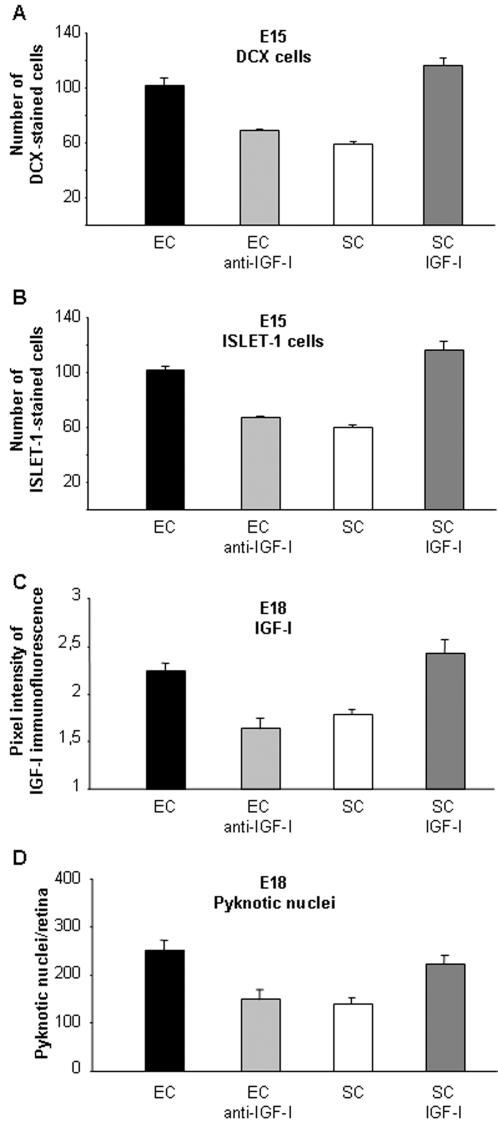
IGF-I is the mediator of maternal enrichment effects on retinal development in the fetus. Number of DCX (A) and of ISLET-1 (B) positive cells in the outer retinal layers of EC, anti-IGF-I EC, SC and IGF-I SC rats at E15. For both (A) and (B), one-way ANOVA showed an effect of the treatment (*p*<0.05). A difference was found between EC and SC, between EC and EC anti-IGF-I and between SC and SC IGF-I groups (*p*<0.05, Post-hoc Tukey test). Neither EC anti-IGF-I and SC groups nor EC and SC IGF-I groups were instead found to differ between each other. (C) Quantitative analysis of IGF-I immunofluorescence intensity in the RGC layer of EC, anti-IGF-I EC, SC and IGF-I SC rats at E18. (D) Pyknotic cell number of EC, anti-IGF-I EC, SC and IGF-I SC rats at E18. After treatment with anti-IGF-I, levels of IGF-I expression and number of pyknotic profiles in the RGC layer of EC fetuses were lowered to those of SC rats while, after chronic IGF-I protein infusion, levels of IGF-I expression and the number of pyknotic profiles in the RGC layer of SC fetuses were enhanced up to those of EC rats. For both (C) and (D), one-way ANOVA showed an effect of the housing treatment (*p*<0.001). A difference was found between EC and SC, between EC and EC anti-IGF-I and between SC and SC IGF-I groups (*p*<0.05, Post-hoc Tukey test). Neither EC anti-IGF-I and SC groups nor EC and SC IGF-I groups were instead found to differ between each other. The bars indicate s.e.m.

To assess whether maternal IGF-I was implicated in the effects of EE on the fetus, we administered, from E10 until E18, a chronic infusion of anti-IGF-I antibody to EC pregnant rats and an infusion of IGF-I protein to SC pregnant rats. We first quantified the number of DCX and Islet-1 positive cells in the fetuses of both experimental groups at E15, i.e. the age at which a difference in the number of migrating cell was detected between SC and EC embryos. We found that the number of DCX and that of Islet-I positive cells was significantly lower in E15 EC embryos treated with anti-IGF-I than in EC untreated animals, and it did not differ from that of SC embryos at the same age (Fig. 5*A, B*). On the contrary, the number of DCX and Islet-I positive cells was significantly higher in E15 IGF-I-treated SC embryos than in SC untreated animals (Fig. 5*A, B*). The effect of IGF-I administration was comparable with that induced by EE: the number of DCX and Islet-I positive cells of E15 IGF-I-treated SC embryos did not differ from that of E15 EE embryos (Fig. 5*A, B*). Thus, IGF-I administration to SC pregnant rats mimicked EE effects on the maturation of retinal progenitors, while anti-IGF-I antibody infusion completely blocked the EE effects.

We then analyzed RGC death levels and IGF-I expression in E18 fetuses of both experimental groups. Treatment with anti-IGF-I totally prevented the IGF-I increase induced by EE in fetal expression in the retina (Fig. 5*C*) and lowered the number of RGC pyknotic profiles in E18 EC fetuses to that found in SC (Fig. 5*D*). IGF-I infusion, on the contrary, was sufficient to induce in SC animals all the reported changes induced by enrichment, i.e. a pronounced increment of RGC layer IGF-I expression and of pyknotic profiles' number (Fig. 5*C, D*).

Although we did not perform a quantitative analysis of IGF-I levels due to the extremely low doses at which the protein is present in the embryonic retinas, taken together our results strongly suggest that IGF-I is a key regulator of the time-course of natural RGC death.

### The influence of maternal enrichment on fetal development is not restricted to the retina

**Figure 6:**
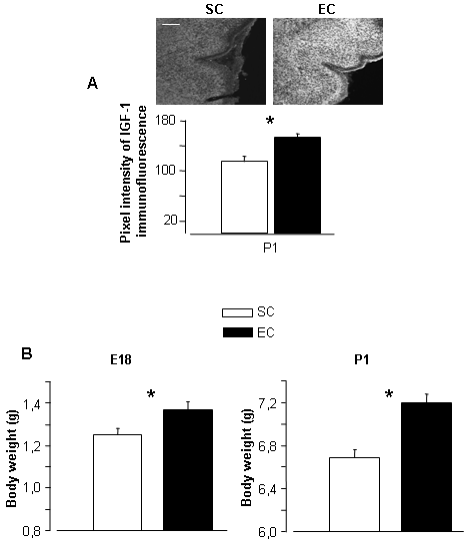
(A) Increased IGF-I levels in the cerebellum of EC rats. Coronal sections through the cerebellum: IGF-I immunoreactivity is low in cerebellar cells of SC rats, while the cerebellar cells of EC rats show a strong IGF-I staining. Scale bar: 50 µm. Graph: quantitative analysis of the pixel intensity of IGF-I immunofluorescence reactivity showed higher levels in the cerebellum of EC (black) compared with SC (grey) rats at P1 (Mann-Whitney rank sum test, *p*<0.05). The bars indicate s.e.m. (B) Prenatal enrichment increases body weight. The weight of EC fetuses was 10% greater at E18 (n = 19 for EC and n = 15 for SC), and 8% greater at P1 (n = 64 for EC and n = 49 for SC). Two ways ANOVA of rat weights for different environmental conditions and ages showed a significant effect of age (*p*<0.001) and environmental housing condition (*p*<0.001). Mann-Whitney rank sum test with Bonferroni correction revealed a significant increase in EC animals body weight compared with SC rats at both E18 and P1 (*p*<0.001 in both cases).

In order to better characterize the influence of maternal enrichment on global fetal development, we investigated whether any maturational changes between EC and SC subjects were detectable also out of the retina. Since IGF-I is known to regulate cerebellar maturation [22] and is a strong modulator of fetal growth [23], we analyzed IGF-I levels in the cerebellum and measured the body weight of EC and SC rats at different ages. Enriched animals had a marked increase in cerebellar IGF-I protein expression compared with SC rats (Fig. 6*A*) and their weight was increased by ∼10% at both E18 and P1 (Fig. 6*B*). It is worth noting that administration of anti-IGF-I antibody to enriched pregnant rats reported fetal weight to control values (EC fetuses = 1,37±0,04 g ; EC anti-IGF-I fetuses: 1,15±0,05 g; Mann-Whitney Rank Sum Test, *p* = 0,005). The increase in cerebellar IGF-I levels and body weight suggests that maternal enrichment during pregnancy affects the global development of the fetus.

## Discussion

Our results show that the development of the nervous system is sensitive to environmental stimulation during prenatal life. The effects of the environment on fetal development are mediated by the mother: enriched environmental conditions provided to the mother during pregnancy affect retinal development by controlling fetal growth factor levels and result even in an increased somatic growth.

### Retinal development is sensitive to prenatal environmental stimulation

We previously demonstrated that EE from birth promotes the maturation of the visual cortex [2]–[4], [24] and accelerates the postnatal development of the retina [4]. The finding that the effects of EE on visual system development are present in the retina is particularly relevant, since this structure has been classically thought to be resistant to experience-dependent alterations. Here we extend this finding, showing that the environmental influence on the visual system development occurs also during prenatal life. Indeed, we report that the maternal environment can profoundly affect the development of the retina in the embryo by anticipating structural processes which are critical for retinal maturation, such as the migration of neuronal progenitors and the time-course of natural cell death. Until now, a shift in the time-course of retinal development has been observed only in mice with natural genetic mutations that affect the rate of natural cell death [25]. This is the first case in which the environment is reported to accelerate the retinal pattern of cell migration and cell death, cardinal features of visual system development. The precise assembly of neuronal circuits requires a tight control of a correct number of pre- and post-synaptic neurons forming synaptic connections. The earlier time-course of RGC death induced by maternal environmental enrichment may be determinant for an accelerated maturation of intra-retinal circuits and sculpting of the retinofugal projections.

### IGF-I: a key player in maternal enrichment effects on retinal development

The effects we found on retinal development seem to be dependent on IGF-I, as suggested by the increased expression we found in EC pregnant rats and in their offspring. Although a rigorous analysis of IGF-I levels in the retinas of SC and EC embryos would require quantitative assays which could hardly be performed because of the very low quantity of IGF-I protein that can be obtained from the embryonic retinas, a strong indication of the IGF-I involvement in the accelerated retinal development induced by prenatal enrichment comes from the results of our artificial manipulations of IGF-I levels in SC and EC pregnant rats. Indeed, we showed that neutralization of IGF-I abolished the action of maternal enrichment on the migration of neuronal progenitors and RGC pyknosis, and that these effects were accompanied by a marked reduction of IGF-I expression in the EC retinas. On the other hand, chronic IGF-I infusions during late pregnancy were sufficient to induce in SC animals all the reported changes elicited by EE on retinal development. Therefore, we think that our results indicate that IGF-I is the mediator of the effects of prenatal enrichment on retinal development. IGF-I receptors are present in the retina [26], [27] and are expressed in a developmental manner [20], [28]. Moreover, it is known that IGF-I has a central role in building the cytoarchitecture of the retina [19], promoting early cell proliferation, differentiation [20] and migration [29], [30]. Therefore, it is possible that alterations in the expression of IGF-I may affect naturally-occurring developmental cell death in the RGC layer. More specifically, it is conceivable that increased levels of retinal IGF-I induced by enrichment may enhance the proliferation of progenitors, which in turn would anticipate the time course of their differentiation, migration and cell death. Such a model is supported by our results showing that the numbers of retinal cells expressing double-cortin and positive for Islet-I were higher in EC compared to SC embryos at E15, suggesting that an earlier time course of cell migration resulted to the anticipated dynamics of RGC death observed in EC animals.

Since Islet-I is a specific marker for ganglion and cholinergic amacrine cells, we can not exclude the possibility that IGF-I could have exerted a parallel action on both RGCs and amacrine cells. On the other hand, we think that one indirect effect of IGF-I on RGCs' development through the influence on displaced amacrine cells is unlikely. Indeed, it is well known that the development of amacrine cells occurs later than that of RGCs [31], which are instead the first class of retinal cells to differentiate, and we found an increased IGF-I expression in the maturing RGC layer of EC rats at a developmental age when displaced amacrine cells are only a minority of the total cell population.

### Concluding remarks

We have recently demonstrated that EE from birth determines a marked acceleration of visual system development, detectable at the functional level, with a precocious maturation of retinal and cortical acuities, and at the molecular level, with increased levels of BDNF in the retina and of BDNF and IGF-I in the visual cortex [2], [4], [32]. The most precocious effects elicited by EE were observed at postnatal ages preceding eye opening, and have been hypothesized to depend on differential maternal stimulation received by pups in different environmental conditions. Indeed, higher levels of maternal care have been shown to be provided to EC compared with SC pups [3].

The findings reported in the present paper demonstrate that the acceleration of visual system development induced by EE can start even before the age of birth. Therefore, we propose a model in which three distinct temporal phases during pup development are differently controlled by the richness of the environment. In the first phase, maternal enrichment during pregnancy affects IGF-I expression in the offspring RGC layer, resulting in an accelerated retinal development. Subsequently, enhanced maternal care levels in EC provide to the developing subject a robust tactile stimulation which can induce higher levels of BDNF in the retina and visual cortex, and a precocious eye opening. Finally, when pups begin to actively explore the surroundings, the complex sensory-motor stimulation provided by EC may directly influence their visual system development, contributing to further accelerate the maturation of visual acuity. The effects present in the three phases occur sequentially, but it is conceivable they are causally linked together, i.e. each phase might act as a trigger for the successive one(s). These studies suggest that the influence of environment on the development and plasticity of visual system is due not only on changes in the levels of sensory visual stimulation, but mostly on factors activated even in the absence of vision.

The finding that maternal enrichment during pregnancy results in an increase of IGF-I, which is essential throughout gestation and is a key player of fetoplacental growth [22], [33], [34], could have a bearing in orienting clinical research in the field of prenatal therapy.

## Materials and Methods

### Animal treatment

All experiments were performed on Long Evans hooded rats in accordance with the Italian Ministry of Health guidelines for care and use of laboratory animals. Rat pups (P1) were anesthetized by hypothermia, young rats (P10) were anesthetized with ether and adult rats by chloral hydrate or avertin (1 ml/100 g body weight).

### Rearing environments

#### Enriched Condition (EC)

Consisted of a large cage (100×50×82 cm) with three floors linked by stairs, containing several food hoppers, two running wheels and differently shaped objects (toys, tunnels, shelters) that were repositioned and/or substituted with others once a week. Every cage housed at least 6 adult females and one male. The male was removed after 7 days. With this procedure, the whole pregnancy occurred in the enriched condition.

#### Standard Condition (SC)

Consisted of a standard cage (40×30×20 cm) housing a maximum of 3 animals (two females and one male). The male was removed after 7 days.

In both environmental conditions food and water were available *ad libitum*.

To obtain timed pregnant rats we used the following procedure: a male was put with the females from 4.00 p.m. until 9.00 a.m. of the following morning, in either SC or EC. The latter calendar day was called embryonic day (E) E0, the first day of gestation.

The day of parturition (i.e. first 24 hours after delivery) has been referred to as P0.

### Histology

To take the eyes from E15 and E18 embryos, pregnant dams were anesthetized with chloral hydrate, perfused through the hearth with 4% paraformaldehyde in 0.1 phosphate buffer (pH 7.4) and the embryos were removed after surgical hysterotomy. To take the eyes from P1 pups, rats were perfused through the hearth with 4% paraformaldehyde in 0.1 phosphate buffer (pH 7.4). The eyes of E18 and P1 rats were fixed in 4% paraformaldehyde for 24 h. Retinas (E18: n = 19 for EC and 10 for SC; P1: n = 25 for EC and 12 for SC; retinas derived from at least two litters per experimental group) were then dissected from the eyes, flattened on gelatinized slides and fixed with 2,5% glutaraldehyde and then with formalin-ethanol solution (1∶9). Whole-mounted retinas were stained with cresyl violet (0.1%). The number of pyknotic profiles was counted following a “blind procedure“ in the RGC layer of 60 fields (80×80 mm) per retina on average, uniformly distributed across the retinas. The proportion of retina sampled in this way ranged from 2.1 to 13.8%. Pyknotic cells were counted at 100× magnification using a Zeiss computerized microscope (software, Stereo Investigator, Microbrighfield). Pyknotic cells were identified by the presence of darkly and uniformly stained nuclei, sometimes fragmented. When two or more fragments were seen within a cell diameter distance from each other, they were counted as a single pyknotic cell. Total number of pyknotic cells per retina was estimated by multiplying the average number of cells per field times the ratio of the total area of each retina to field area. For microglial cell number analysis, the *Griffonia simplicifolia* lectin labeling was performed on whole-mount retinas of P1 rats (EC, n = 6; SC, n = 6). Retinas were incubated overnight in B4 isolectin biotinylated (0.025 mg/ml, Sigma). Bound lectin was revealed by ABC kit (Vector) and nickel-enhanced diaminobenzidine (DAB) reaction. Microglial cells were counted at 100× magnification. The total number of microglial cells per retina was estimated using the same counting procedure described before.

### Analysis of natural cell death with the Tunel method

Since the newborn rat ganglion cells are stacked in a pseudostratified fashion and it is difficult to detect the border between the RGC layer and the inner plexiform layer [16], we cannot exclude the possibility to have counted pyknotic profiles in both of these layers with the cresyl violet staining procedure. Therefore, we repeated the analysis on natural cell death using the Tunel method, analyzing also levels of apoptosis at E20. In this analysis, counterstaining of retinal coronal sections with the nuclear marker TOTO (see below) allowed us to clearly visualize the position of fragmented nuclei in different retinal layers.

To detect DNA fragmentation in RGC layer dying cells, terminal deoxynucleotidyl transferase mediated dUTPNick.End Labeling (TUNEL) technique was employed, using a commercially available kit (DeadEnd™Fluorometric Tunel System, Promega). Eyes were immersion-fixed in 4% paraformaldehyde, cryoprotected in 30% sucrose and embedded in Tissue-Tek. Retinal sections of 18 µm were cut using a cryostat and collected in serial order through the entire retina. After treatment with proteinase K (20 µg/µl) to dissociate proteins from DNA, sections were incubated (1 h at 37°C) with “the Tunel reaction mixture”, containing the TdT enzyme and Nucleotide mix with fluorescein-12-dUTP. Retinal sections (n = 4 SC and EC rats for each age) were then counterstained with TOTO-3 iodide (Molecular Probes) to visualize the different cell layers, and rinsed in PBS. In the negative controls, which never gave any significant staining, the Tunel reaction mixture was omitted. Tunel-positive cells were counted with “a blind procedure” by the use of a 40× objective, in the RGC layer of 10 equally spaced sections per retina. Each retinal section was completely sampled. The total number of cells per retina displaying fragmented nuclei in the RGC layer was calculated by multiplying the average number of labeled cells per section times the total number of retinal sections. The morphological appearance of retinal layers was indistinguishable between EC and SC rats.

### Analysis of RGC number

We analyzed RGC number in EC and SC rats at P1 (n = 10 for both SC and EC) and in adulthood. We estimated this number in cresyl violet-stained whole mounts retinas, excluding cells with a diameter of less than 8 µm with a darker stained nuclei, a cytological characteristic of displaced amacrine cells [16], [35] and small cells with mottled nuclei, described to be glial cells [36].

For analysis of the final number of retinal ganglion cells in adult EC rats, 7 EC and 3 SC rats (P>45) were deeply anesthetized with avertin. The left optic nerve was retro-orbitally transected mechanically with thin surgical forceps. All animals were killed 1 month after surgery, when most (about 95%) of RGCs were lost in the eye ipsilateral to the lesioned optic nerve [35]. The relative proportion of displaced amacrine cells and RGCs in enriched adult rats was not known, so we counted the total number of RGCs in retinas ipsilateral (IR) and contralateral (CR) to the lesioned optic nerve. These numbers were estimated using the same counting procedure described before. Since amacrine cells are not affected by optic nerve lesion [16], RGC number was calculated by subtracting the number of displaced amacrine cells (counted in IR) from the number of cells counted in CR.

### Determination of IGF-I concentration in the maternal milk

Milk samples were collected from P1 and P10 suckling pups. Pups (P1, n = 15 for both EC and SC; P10, n = 6 for both SC and EC groups) were killed between 9 and 10 a.m. through decapitation and the gastric content was quickly removed, weighed and frozen at −80°C until assayed. Milk samples were homogenized with distillate water and centrifuged at 14000 rpm at 4°C for 30 min to separate the whey (infranatant) from the fat (supernatant) and casein (pellet). The whey milk was acid-ethanol extracted to remove IGF-I binding proteins. The concentration of IGF-I was determined by radio immunoassay (RIA) using a commercial kit specific for rodents (DSL-2900, Diagnostic Systems Laboratories, Webster, TX), with a sensitivity of 21 ng/ml.

### Immunohistochemistry

For IGF-I immunostaining, vertical retinal sections (16 µm thick) and cerebellar sections (40 µm thick) were cut using a cryostat and then processed as follows. Sections were permeabilized in 0,3% triton X-100 and incubated in 1∶500 rabbit polyclonal anti-IGF-I antibody (kindly provided by Prof. Ignacio Torres-Aleman). Bound antibody was detected by incubating sections with biotinylated goat anti-rabbit IgG (1∶200, Vector) followed by fluorescein-conjugated extravidin (1∶300, Sigma). The number of animals used for IGF-I analysis in the retinas was: 10 (E15), 7 (E18), 7 (P1), 4 (P10), for EC; 6 (E15), 5 (E18), 7 (P1), 4 (P10), for SC. For the analysis of IGF-I in the cerebellum, 4 animals per group were used.

For doublecortin (DCX) immunostaining, vertical retinal sections were incubated, after a blocking step, in goat polyclonal anti-doublecortin antibody (1∶1000, C-18 Santa Cruz), then exposed to biotinylated rabbit anti-goat antibody (1∶200, Vector lab) followed by fluorescein-conjugated extravidin (1∶300, Sigma). The number of animals used in DCX analysis was: 6 (E15) and 5 (E18) for EC; 4 (E15) and 4 (E18) for SC. For Islet-1 and calbindin immunostaining, vertical retinal sections were incubated, after a blocking step, in mouse anti-Islet-1 antibody (1∶50, DSHB) and rabbit anti-calbidin antibody (1∶1000, Swant), respectively. Immunoreaction was detected using biotinylated horse anti-mouse or biotinylated goat anti-rabbit antibody (1∶200, Vector lab) followed by fluorescein-conjugated extravidin (1∶300, Sigma). The number of animals used in Islet-1 analysis was: 6 for E15 EC; 3 for E15 SC. For the analysis of Calbindin, 3 animals per group (E15 EC and SC) were used.

### Counting of DCX, Islet-1 and calbindin labeled cells

Images were acquired using an Olimpus Optical confocal microscope at 20× magnification (zoom 1.5, field 460×460 µm, acquired at 1024×1024 pixels). Settings for laser intensity, gain, offset and pinhole were optimized initially and held constant through the study. For each retina, at least 4–5 sections (16 mm thick) taken at the level of the optic nerve head were acquired, imaging four fields per each section. The collected images of the retina were imported to the image analysis system MetaMorph and used to count the number of labelled cells in the neuroblastic layer. All acquisitions and counting analysis were performed blind to the rearing and experimental condition. To identify whether the migrating cells were also positive for ISLET-1, a double labeling for both DCX and ISLET-1 was performed in SC and EC retinas at E15. Images were acquired at 60× magnification, zoom 2.5, field 92×92 µm, 1024×1024 pixels.

### Chronic infusions of anti-IGF-I antiserum or IGF protein to pregnant rats

Timed pregnant rats were reared in either SC or EC since the start of gestation. At E10, anti-IGF-I antiserum or IGF-I protein were infused to EC and SC pregnant rats, respectively. It has been reported that the anti-IGF-I antibody has <1% cross-reactivity with either insulin or IGF-II, as determined by competition with ^125^I-IGF-I (for reference, see ref. 37). Infusions were done through implantation of a subcutaneous osmotic minipump (Alzet; anti-IGF-I infusion: 20% in saline; IGF-I protein infusion:1 µg/µl; infusion rate: 0.25 µl/h in both cases) placed in the back of the animal in the scapula [38]. Qualitative observations performed during both daytime and during the dark phase of the daily cycle revealed an apparently normal behavior of implanted pregnant rats. In particular, EC pregnant rats were frequently observed to use the running wheel. At E18, pregnant rats were perfused transcardially and their embryos were removed through surgical hysterotomy. The eyes of the embryos were removed, fixed and processed for DCX and ISLET-1 positive cell number in the retinal outer layers (DCX: n = 7 animals for EC, n = 5 animals for SC; ISLET-1: n = 7 animals for EC, n = 5 animals for SC), RGC apoptosis analysis (n = 13 animals for EC, n = 20 animals for SC) and IGF-I expression levels (n = 7 animals for EC, n = 5 animals for SC), as previously described. Examination of histological brain sections revealed no signs of malformations or gross morphological abnormalities in both anti-IGF-I and IGF-I embryos.
